# In situ exogenous alpha-synuclein aggregates inhibit murine ventricular voltage-gated inward sodium and outward potassium currents

**DOI:** 10.1177/1877718X251365239

**Published:** 2025-08-28

**Authors:** Bonn Lee, Shiraz Ahmad, Charlotte E Edling, Hugh R Matthews, Christopher L-H Huang, Fiona EN LeBeau, Kamalan Jeevaratnam

**Affiliations:** 1 School of Veterinary Medicine, Faculty of Health and Medical Science, University of Surrey, Surrey, UK; 2 Physiological Laboratory, University of Cambridge, Cambridge, UK; 3 Department of Biochemistry, University of Cambridge, Cambridge, UK; 4 Biosciences Institute, Faculty of Medical Sciences, University of Newcastle, Newcastle upon Tyne, UK; 5 The Medical School, Framlington Place, Newcastle upon Tyne, UK

**Keywords:** alpha-synuclein aggregates, cardiac alpha-synuclein, loose patch clamp, Parkinson's disease, ventricle

## Abstract

**Background:**

Alpha-synuclein is associated with neurodegeneration in Parkinson's disease (PD). Recent studies have increasingly recognized incidences of cardiac complaints in PD patients. In particular, the occurrence of arrhythmias in PD patients may indicate potential electrophysiological alterations in the heart. Alpha-synuclein aggregates have been known to have disruptive effects on cell membranes. However, the effect of alpha-synuclein on the heart and sympathetic neuronal tissues remains unknown.

**Objective:**

This study investigated the electrophysiological effects of alpha-synuclein aggregates in myocardium and cardiac sympathetic nervous system, potentially reflecting cardiac electrophysiological alteration in PD.

**Methods:**

We measured the *in situ* sodium and potassium currents from murine ventricular myocardium and stellate ganglia using the loose patch clamp technique. The tissues were exposed to bioactive alpha-synuclein aggregates, and currents were measured under three different conditions: baseline, alpha-synuclein treatment, and wash out.

**Results:**

The experiments showed that alpha-synuclein aggregates altered the maximum cardiac sodium current (*I*_Na(Max)_) (ANOVA, p < 0.008) and affected its gating properties for channel activation (ANOVA *F*_2,54_ = 6.408, *p* = 0.003) and inactivation (*F*_2, 67_ = 6.32, *p* = 0.003). The alpha-synuclein aggregates also reduced the maximum outward potassium current (*I*_K(Max)_) during channel activation (*F*_2, 77_ = 6.02, *p* = 0.002). However, the alpha-synuclein aggregates did not affect the ionic currents in the stellate ganglia.

**Conclusions:**

Our results demonstrate that extracellular alpha-synuclein aggregates can inhibit ventricular but not stellate ganglion ionic currents, suggesting a differential sensitivity between the myocardium and the stellate ganglia, and indicating a cardiac-specific toxicity of alpha-synuclein on cardiac electrophysiology.

## Plain language summary

Alpha-synuclein is the pathological hallmark of Parkinson's disease (PD). There is an increased recognition of incidences of heart-related complaints in PD patients. In particular, arrhythmias have been associated with PD. This may indicate potential pathological changes in the heart. Alpha-synuclein has been known to be toxic to mammalian cells. However, its effect has not been tested in the heart and heart-connecting nerve tissues. Therefore, we investigated the effect of alpha-synuclein aggregates on the heart and the heart-connecting nervous tissues. The heart and stellate ganglia conveying nerves to the heart were exposed to bioactive alpha-synuclein aggregates, and the ionic current in these tissues were measured. The results showed that alpha-synuclein aggregates reduced the size of the inward sodium current in the heart. However, the alpha-synuclein aggregates did not affect the ionic currents in the stellate ganglia. Our results revealed that alpha-synuclein aggregates can alter cardiac ionic currents, which imply that a cardiac-specific toxicity of alpha-synuclein aggregates on cardiac electrophysiology in PD.

## Introduction

Alpha-synuclein is a 140 amino acid protein that was first identified in 1988.^1^ About ten years later, a positional cloning genomic study exploring kindreds with familial Parkinson's disease (PD) revealed that alpha-synuclein (a-SYN) mutation was associated with PD.^2^ Building on this advance, Spillantini et al. (1998) showed that alpha-synuclein is the major constituent of Lewy bodies (LB), the pathological hallmark of neurodegeneration developing in PD, Lewy body dementia, and multiple system atrophy.^3,4^ Multiple, including monomeric, dimeric, oligomeric and fibrillar, forms of alpha-synuclein, and protein misfolding associated with alpha synuclein aggregation, can present in Lewy bodies.^5^ The PD-related mutations, A53T, A30P, and E46K on the alpha-synuclein gene, are known to accelerate the oligomerization, which are established as risk factors for early onset PD.^6^ Among the various forms of a-SYN, the oligomeric form is known to be toxic to mammalian cells *in vitro* and *in vivo*,^7^ directly disrupting cellular structure and inducing cell death. In contrast, extracellular alpha-synuclein monomer interferes with neuronal cell proliferation and reduces cell survival rate via the Akt signaling pathway.^8^ The physiological role of a-SYN remain unclear; however, extensive research has indicated that alpha-synuclein involves neurotransmitter release, particularly in the regulation of pre-synaptic vesicle release.^9–12^ Alpha-synuclein is enriched in neuronal presynaptic terminals.^1^ Recent studies have investigated the concentration of a-SYN in the systemic circulation, including cerebrospinal fluid,^13^ plasma^14^ and serum,^15^ focusing on its potential as a biomarker for the diagnosis of PD. Alpha-synuclein in systemic circulation can be detected in an oligomeric or phosphorylated form.^13,16^ Interestingly, a recent study demonstrated that patients with PD exhibit elevated levels of phosphorylated a-SYN deposition in the kidney, suggesting that systemic circulation of a-SYN may contribute to the pathogenesis of PD and other LB-associated disorders.^17^ Clinical studies with PD patients revealed that the level of circulating a-SYN correlated with PD severity. However, the segregation of this index between PD patients and non-patients is still unclear. Importantly, circulating a-SYN can interact with cell membranes, adopting amyloid conformations.^18^ Specifically, cell damage mediated by membrane-bound a-SYN, such as increased reactive oxygen species or reduced mitochondrial activity, correlates with its close structural interaction with the lipid bilayer of neuronal cell membranes.^19^

In the central nervous system (CNS), a-SYN aggregates have been known to impair neuronal electrophysiological activity.^20–22^ For example, neuronal excitability can be affected by a-SYN aggregates in hippocampal neurons via interaction with normal cellular prion protein (PrP^C^) and NMDA receptors.^21^ Neuronal microglia can interact with a-SYN aggregates through cell-immune receptors, leading to an increase of Kv1.3 channel activity. Additionally, there is also evidence that a-SYN aggregates can affect the electrophysiology of cellular membranes, even in non-neuronal cells. It has been demonstrated that the lipid bilayer membrane bounding a-SYN aggregates may form ionic channel-like structures, affecting the current flow of the membrane in yeast or synthetic biomembranes.^18,23–25^

Cardiovascular abnormalities have been increasingly recognized in PD patients.^26,27^ Cardiac dysautonomia is now an established feature of PD.^28^ The occurrence of arrhythmias and sudden cardiac death (SCD) in PD patients, potentially reflecting cardiac electrophysiological alterations, has become a growing concern.^27,29^ In this context, we focus on recent findings related to cardiac a-SYN. Previous studies have demonstrated that a-SYN aggregates indeed develop in PD patients’ hearts, specifically in the nerve and fat tissues of the epicardial region.^29–31^ Furthermore, one report has correlated high plasma circulating a-SYN with cardiac sympathetic denervation, rather than with nigrostriatal degeneration in the brain, as reflected in MIBG heart-to-mediastinum (H/M) ratios.^32^ Nonetheless, there remains a lack of studies directly investigating the effect of a-SYN on cardiac tissue.

Stellate ganglia (SG) provide sympathetic autonomic input to the heart thereby mediating cardiac rhythmic control.^33^ The SG are involved in cardiac dysautonomia and disorders such as arrythmias, chronic heart failure, and SCD.^34^ Lewy body pathology has been known to develop in SG.^35^ We hypothesized that the a-SYN aggregates developing in the SG may contribute to cardiac dysautonomia and arrhythmogenic alterations associated with PD. Therefore, SG tissue was included in the analysis, alongside myocardium, to investigate the electrophysiological effect of a-SYN aggregates.

The loose patch clamp technique measures the transmembrane current flow into a large-extracellular electrode that is loosely attached to tissue surface. Due to the nature of the loose seal, the area under the pipette is amendable to external pharmacological manipulations. Additionally, the loose patch technique has been previously adopted to investigate ventricular tissue currents *in situ*.^36^ Moreover, in contrast to the limitation that whole-cell patch clamp captures the electrical activity of a single cardiomyocyte, loose patch clamp recordings can encompass an entire cellular system involving multiple cardiomyocytes within an organizational framework.^37^ In this regard, the loose patch clamp investigation is a well-suited tool to detect potential changes in membrane ionic conductance driven by alpha-synuclein aggregates in the heart and stellate ganglia tissues.

This study aims to explore the a-SYN aggregates-driven changes in ionic currents in ventricular and stellate ganglia tissue. The loose patch clamp technique can reveal whether there is an alteration in sodium or potassium current flow through the membrane. We hypothesized that alpha-synuclein aggregates may alter the electrophysiology of the myocardium and stellate ganglia, potentially reflecting cardiac electrophysiological alterations in patients with PD.

## Methods

### Animal experiments and ethics

Male C57BL/6J wildtype mice were supplied from Charles River Laboratory UK (Margate, Kent, UK) and were maintained in the Biomed Research Facility at the University of Surrey under controlled conditions (ambient temperature 23 ± 2°C, 12-h light cycle) with food and water supply *ad libitum*. Animals were given one week for acclimatization to the housing conditions before experiments were conducted. All animal treatment and procedures were approved by the Animal Welfare Ethical Review Body of the University of Surrey (NASPA-1819-25 Amend 1). All the procedures were performed conforming to the guidelines from Animal Scientific Procedures Act 1986 (UK) and NIH Guide for the Care and Use of Laboratory Animals.

### Tissue dissection for ventricle and stellate ganglia

Mice were euthanized by Schedule I cervical dislocation (as per the Animal Scientific Procedures Act 1986 (UK)). The abdominal cavity was opened, and the thoracic cavity approached via the diaphragm. The heart and stellate ganglia with the pulmonary cassettes and vessels and immediately placed in ice-cold Krebs-Henseleit (KH) solution (108 mM NaCl, 25 mM NaHCO_3_, 4 mM KCl, 1.2 mM KH_2_PO4, 1 mM MgCl_2_, 1.8 mM glucose, and 2 mM sodium-pyruvate, bubbled with carbogen gas and pH adjusted to 7.4) After isolating the heart, the thoracic cavity was placed in an ice-cold physiological solution (92 mM NaCl, 2.5 mM KCl, 30 mM NaHCO_3_, 1.25 mM NaH_2_PO_4_, 20 mM HEPES, 25 mM glucose, 10 mM MgCl_2_, 0.5 mM CaCl_2_; bubbled with carbogen gas; pH adjusted to 7.4) and moved to the loose patch clamp room. The SGs were excised using micro scissors and tweezers by a previously described technique.^38^

### Langendorff perfusion and excision of ventricle

The heart tissue was placed on a petri dish filled with ice-cold KH solution, then the aorta was canulated with a 22-guage stainless steel canulae and the tip was sutured with 5-0 braided silk thread. The canulated heart was placed in a Langendorff perfusion system and underwent retrograde perfusion under constant flow (2.1 mL/min). The heart was placed in an ice-cold KH solution for the dissection, then the right ventricle was excised. The materials used for Langendorff perfusion to perform a loose patch clamp were previously described.^39^ After perfusion, the right ventricle was excised from the heart and mounted on a Sylgard plate (SYLGARD 184 Curing agent, Cat# 761028, Sigma-Aldrich) using insect pins.

### Stellate ganglia tissue preparation for the loose patch clamp

Right stellate ganglia were used for the loose patch clamping. The SG tissue then was incubated in a physiological solution containing 2 mg/ml collagenase-P (Cat# COLLAP-RO, Roche Ltd, Indianapolis, IN, USA) at 36°C for 10 min. This procedure serves to remove the epineural and perineurial barriers that insulate tissue from the voltage clamp, thereby allowing the ganglionic cells to be accessed for patch-clamping (Supplemental Figure 1). Then the SG preparation was mounted on a Sylgard plate with standard insect pins, and the plate was placed in the patch clamp chamber. The recording chamber was filled with physiological solution.

### Loose patch clamp pipette fabrication

Pipettes for the loose patch clamp was pulled from borosilicate glass capillary tubing (Cat# GC150-10**;** Harvard Apparatus, Cambourne, UK) by utilizing a pipet puller apparatus (Cat# PC-10 Narishige, Tokyo, Japan) as previously described.^39^ The pipette tips with an internal diameter of 18–26 μm after polishing were selected for experimental use, then mounted on the microelectrode holder (Cat# Q45W-B15P). incorporating an Ag/AgCl half-cell connected to the headstage.

### Pipette positioning and forming a loose seal onto the tissue surface

By manipulating the syringe, the distal 1/3 of the pipette was filled with physiological solutions. Then the pipette was lowered perpendicularly to the membrane of the tissue using a fine vertical manipulator (Prior Scientific Instruments, Cambridge, UK). Under a loupe, the pipette was positioned onto the tissue. The pipette tip was advanced until it contacted the tissue surface. This contact with the tissue surface was recognized when it elicited a rise in resistance at the pipette tip, observed by a deflection in oscilloscope trace. Then, the seal resistance (*R*_seal_) was compensated by adjusting the corresponding resistance in the compensating bridge circuit. The seal was subsequently stabilized by applying negative pressure using a syringe. The chamber was grounded at reference potential to complete the circuit. Ag/AgCl electrodes were used to provide a reversible electrical connection between the patch clamp chamber and the electronic circuit.

### Loose patch clamp recording

The current flow across the patch of the membrane drawn into the pipette could be measured by the electrode in the pipette, relatively to the grounded reference potential of the patch clamp chamber. The potential across the membrane within the patch then corresponds to the cell resting membrane potential (RMP) prior to application of the pulse protocols. Then, the pulse protocols clamped the voltage of the fluid within the pipette through the sequence of commands potentials. This achieves the required changes in potentials across the membrane within the patch. Since the voltages are applied from the extracellular rather than the intracellular space, a negative voltage step causes hyperpolarisation and a positive step causes depolarisation of the membrane patch relative to RMP. Membrane potentials in this manuscript are thus described relative to the RMP, and imposed voltage changes expressed as changes in intrapipette potential.

### Alpha-synuclein aggregates

Alpha-synuclein aggregates were used to investigate the effects on the ventricle and SG tissue membranes. Due to the nature of the loose patched seal, the membrane area under the patch is amenable to the external biochemical environment of the pipette. A bioactive form of Recombinant Human Alpha-synuclein protein aggregates (αSYN; Cat#: ab218819, Abcam, Cambridge, UK) were stored at a −80°C freezer as a stock solution (1μg/μl in PBS, provided from Abcam). The calculated volume was taken from the stock solution, and diluted in physiological solutions at room temperature as a working solution, then gently vortexed. For baseline recording, the patch clamp chamber was filled with KH or physiological solution, then the solutions were gradually replaced with the working solution containing αSYN. The circulating concentration (200 pM) was determined considering the alpha-synuclein levels in the cerebrospinal fluid of Parkinson's disease model animal, referred to Kim et al. (2022).^40^ The effective concentration was set at a concentration proven to impair synaptic transmission in murine brain slices, referred to Diogenes et al. (2012).^21,41^ A 25 min of equilibration time was allowed after replacing the alpha-synuclein containing physiological solution. The solution was used up to maximum 40 mins to prevent potential metabolite accumulation from the tissue preparation.

### Random peptide control

To validate whether the inhibitory effect caused by the alpha-synuclein aggregates was not due to non-specific peptide components, a peptide mixture was used as a control. The peptide mixture was prepared by combining two different peptide sequences in a 1:1 ratio. The sequences for the peptides were as follows: (C-terminal) TTKINMDDLQPSENEDKS (Cat#: BLP-CC003, Alomone labs, Jerusalem, Israel), (C-terminal) LQTFSRPQGSEEAGAGDEEEDM (Cat#: BLP-CC021, Alomone labs). The peptide mixture was dissolved in KH or physiological solution as a working solution at a concentration of up to 1 μM, then was superfused onto the ventricular preparation and incubated up to 40 mins in the loose patch clamp bath.

### Data recording from the loose patch clamp

Electrophysiological recording were made using a custom written software used to deliver voltage clamp steps relative to the RMP.^39^ Currents were sampled at a 50 kHz digital sampling rate and filtered at a DC-10 kHz bandwidth using a 10 kHz Bessel low pass filter. Patches containing sodium channels produced kinetically characteristic inward currents. We investigated their voltage-dependent activation and inactivation properties, as well as the voltage-dependent activation of transient outward potassium currents. The currents obtained were normalized to the area of pipette tip to achieve the current density, following the formula: current density (pA/μm^2^) = current measured (nA) × 1000 /{π × [pipette radius (mm)]}^2^.

### Determination of the channel opening kinetics by curve fitting to the boltzmann equation

Each current-voltage curve was fitted to the Boltzmann equation to ascertain the opening kinetics for voltage-gated ion channels. The sodium ionic currents (*I*) were related to the activating voltage, designated as *V* = *V*_1_, through a Boltzmann function expressed by the equation: *I* = *I*_max_ [1 − 1/ [1 + exp [(*V* − V*)/k] ] ]. The peak current's maximum value is given by *I*_max_, the voltage corresponding to half of the maximal peak current by V*, and the parameter denoting the slope factor associated with the voltage sensitivity of the current by *k*. The slope factor was expressed in millivolts (mV), with a higher value denoting a shallow curve.

### Data handling and statistical analysis

We investigated different areas on the tissue surface with the loose patch clamp. Each patch-clamped site counted as a separate data point (n of 1). When a different heart or SG was isolated, it was counted as an independent experiment. We examined four sites in each tissue. In cases where fewer than four sites were examined due to the size variance of the tissue, the number of sites investigated is denoted in the figure legends. The number of replicates is listed in the figure legends. The statistical significance analysis was performed using R 4.4.0. The Shapiro-Wilk normality test was applied to determine if the data followed a normal distribution. After confirming normality, comparisons were made using Student's t-test for comparing the means. One-way ANOVA, and *post hoc* Tukey honestly significant difference test were used to determine the significance level among multiple unrelated groups.

## Results

### Alpha-synuclein aggregates alter sodium channel activation properties in the ventricle

**Figure 1.**
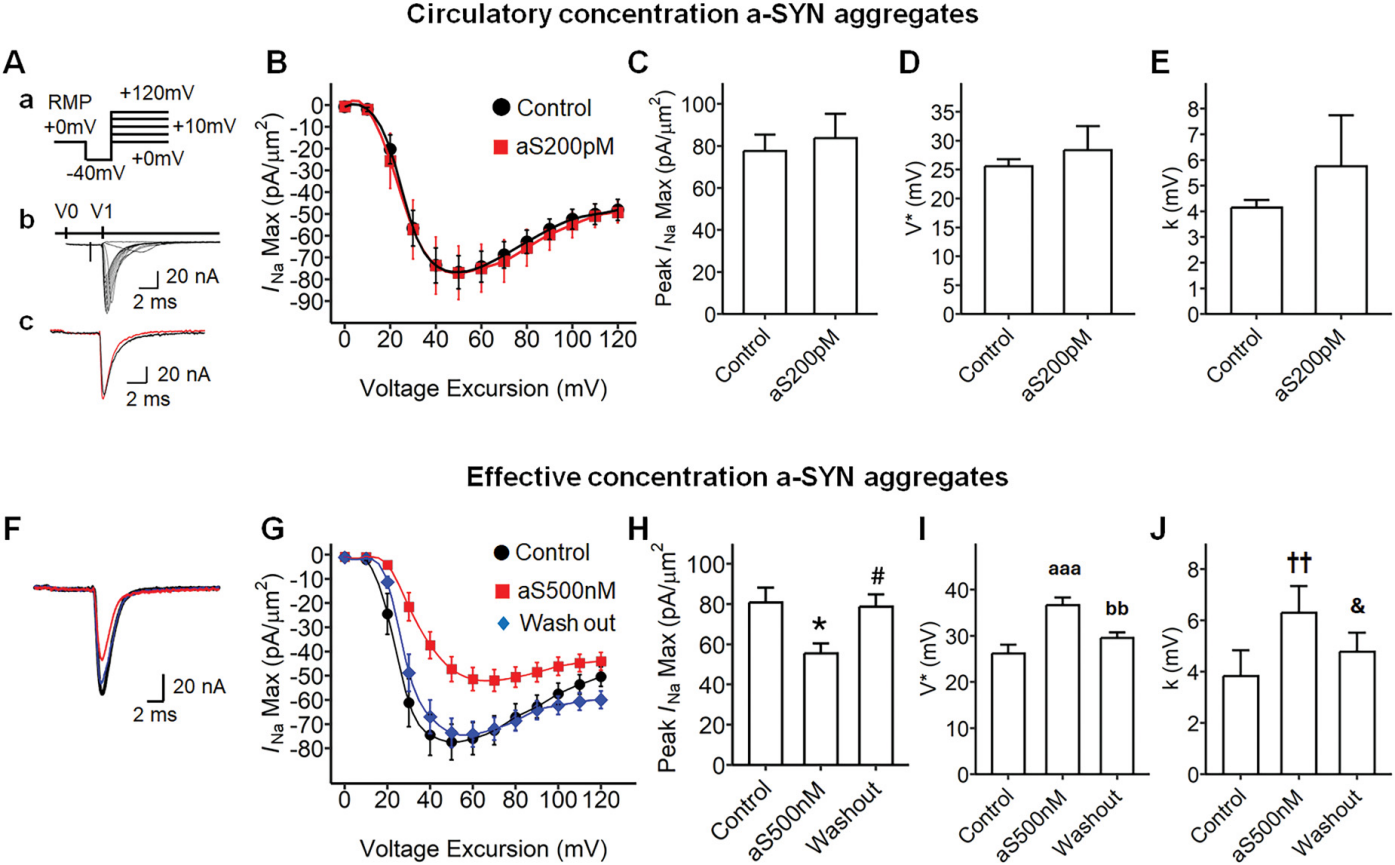
Effect of alpha-synuclein aggregates on the ventricular sodium channel activation properties under loose patch. (Aa) Step pulse protocol to activate sodium currents, RMP resting membrane potential, activation pulse protocol began from the RMP, a 4 ms duration prepulse was applied to the patched area to remove any residual current over the area of the pipette, then 10 ms step pulses were applied to elicit the voltage-gated currents negative 40 mV from the RMP (RMP - 40 mV) and through to positive 120 mV from the RMP (RMP + 120 mV). (Ab) A family of current traces from the pulse protocol in a, V0, −40 mV prepulse at a 1 ms duration; V1, 10 mV step pulses at a 5 ms duration. (Ac) The example current traces for KH control (black) and alpha-synuclein (a-SYN) 200 pM treatment (red). (B) A current-voltage curve of sodium activation for circulatory dose a-SYN, *I*_Na(Max)_ of each maximum current was plotted against the voltage excursion; Control, baseline recording with Krebs-Henseleit (circle, n = 11, from three independent subjects); aS200pM, a-SYN 200 pM treatment (square, n = 8, from three independent subjects). (C) The greatest value in B (*I*_Na(Max)_), control and aS200pM, −77.54 mV and −83.80 mV respectively, no significance. (D) The half-maximal voltage (V) from B, control vs. aS200pM, −77.54 mV and −83.80 mV, no significance. (E) The Boltzmann slope factor (*k*) in B, *k* for control and aS200pM, 4.14 mV and 5.75 mV respectively, no significance. (F) Current trace for a-SYN challenging dose, KH control (Control, black), a-SYN 500 nM treatment (aS500 nM, red), washing out with KH solution after the treatment (Wash out; blue). (G) A current-voltage curve of sodium activation for challenging dose a-SYN, control (circle, n = 13, from six independent subjects), aS500 nM (square, n = 20, from six independent subjects) Wash out (diamond, n = 24, from six independent subjects). (H) The maximum current (*I*_Na(Max)_) in G, ANOVA (*F*_2, 54_ = 5.25, *p* = 0.008), *p = 0.027 Control vs. aS500 nM, ^#^*p* = 0.015 aS500 nM vs. Wash out. (I) The half-maximal voltage (V*) from G, ANOVA (*F*_2, 54_ = 11.47, *p* < 0.0001), ^aaa^*p* = 0.0001 Control vs. aS500 nM, ^bb^*p* = 0.002 aS500 nM vs. Wash out. (J) The Boltzmann slope factor (*k*) in G, ANOVA (*F*_2,54_ = 6.408, *p* = 0.003), ^††^*p* = 0.003 Control vs. aS500 nM, ^&^*p* = 0.041 aS500 nM vs. Wash out. RMP: resting membrane potential.

To investigate the effect of alpha-synuclein on ventricular myocardium, bioactive human alpha-synuclein aggregates were applied to the ventricular tissue preparation via superfusion in KH solution (Figure 1). We first applied a circulatory concentration a-SYN (200 pM) to the ventricular preparation and compared the inward sodium currents during sodium channel activation (Figure 1A-E). A voltage step protocol was applied to elicit membrane depolarization of the ventricle (Figure 1A). the maximum magnitude of the inward sodium current (*I*_Na(Max)_) was plotted against the applied voltage-excursion (Figure 1B and C). The current-voltage curve was fitted using the Boltzmann equation to compare the kinetics of sodium channel activation, and the half-maximal voltage (V*) and the slope factor (*k*) were not significantly different between the KH control and the a-SYN 200 pM treatment (Figure 1D and E). Thereafter, we examined the influence of a higher concentration of a-SYN (500 nM solubilized in KH; Figure 1F-J). The effective concentration previously shown to disturb synaptic transmission.^41^ The protocol for activating voltage-gated sodium current was the same as in Figure 1Aa. Interestingly, the a-SYN 500 nM concentration reduced the maximum inward sodium currents (*I*_Na(Max)_) in the ventricle compared to the control (aS500 nM; Figure 1F and G, red trace). The current was subsequently restored on washout with pure KH solution (Wash out; Figure 1F and G, blue trace). The maximum inward sodium currents (*I*_Na(Max)_), recorded as the voltage excursion around RMP + 50 mV, was significantly reduced in a-SYN 500 nM and recovered in wash out (Figure 1H, *p* = 0.02 Control vs. aS500 nM; *p* = 0.01 aS500 nM vs. Wash out). We then quantitatively analyzed the current-voltage curve among the groups (Figure 1I and J). The half-maximal voltage (V*) was significantly higher in the a-SYN 500 nM, compared to that in control or wash out (Figure 1I, ANOVA *p* < 0.0001, *p* = 0.0001 Control vs. aS500 nM; *p* = 0.002, aS500 nM vs. Wash out). The Boltzmann slope factor (*k*) was significantly increased in the a-SYN 500 nM compared to the control and wash out, suggesting that the a-SYN may impact the sodium channel gating properties (Figure 1J, ANOVA *p* = 0.003, *p* = 0.003 Control vs. aS500 nM, *p* = 0.041 aS500 nM vs. Wash out).

To validate whether the changes observed with the exogenous application of a-SYN were due to the a-SYN itself and not non-specific peptide components, an additional experiment was conducted using different peptides (see Supplemental Material). The sodium channel activation, inactivation, time-dependent channel recovery, as well as the potassium channel activation and inactivation properties, were compared between the ventricular preparations superfused with the peptide mixture. The sodium channel activation, inactivation, time-dependent channel recovery, the potassium channel activation, and inactivation properties were compared between the peptide mixture superfused ventricular preparation (PEP, n = 7, from three independent subjects) and the control (Control, n = 6, from three independent subjects). No significant difference was detected between the control and the peptide mixture superfused tissue (Supplemental Figures 2−6). Thus, it was confirmed that the alteration in the ventricular currents was mediated by the effect of a-SYN. The effective concentration a-SYN aggregates impacted ventricular sodium channel activation by reducing the current at the maximal activating voltage and altering the gating property of the channel.

### Alpha-synuclein effective concentration impacted the sodium channel inactivation in the ventricle

**Figure 2.**
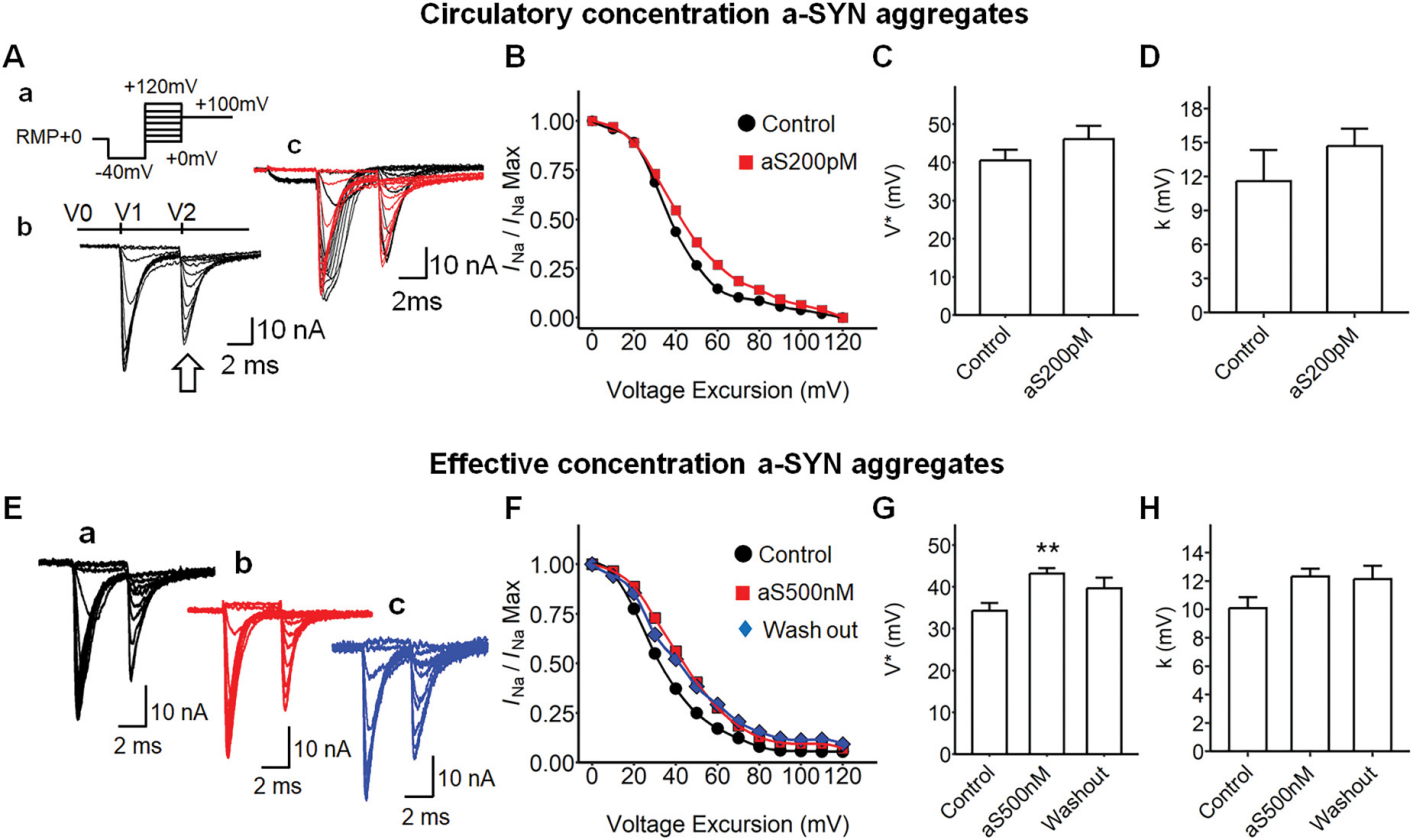
Alpha-synuclein aggregate treatment to the ventricular preparation under loose patch and the measurement of its sodium channel inactivation properties. (Aa) Step pulse protocol, RMP resting membrane potential, a 4 ms duration prepulse was applied to the patched area to remove any residual current over the area of the pipette, then step pulses were applied to elicit the voltage-gated currents negative 40 mV from the RMP (−40 mV) and through to positive 120 mV from the RMP (+120 mV), then the voltage sweep were stopped at RMP + 100 mV. (Ab) A family of current traces from the sodium channel inactivation protocol in Aa, arrow mark, the inward current determining channel inactivation. (Ac) Example traces, arrow mark, the maximum inward sodium current after the activating pulse, V0, −40 mV prepulse at 1 ms; V1, 10 mV step pulses at 5 ms, V2, 100 mV stop pulse at a 10 ms duration; Control (black), alpha-synuclein (a-SYN) aggregates 200 pM in KH (aS200pM, red). (B) The current-voltage curve of sodium channel inactivation. The value of sodium current was normalized to its greatest value at RMP + 0 mV, baseline recording in KH (Control, circle, n = 8, from three independent subjects) vs. aS200pM (square, n = 8, from three independent subjects). (C) The half-maximal voltage (V*) from a Boltzmann equation described with B, −46.12 mV and −40.53 mV, Control vs. aS200pM, no significance. (D) The Boltzmann slope factor (*k*) in B, 14.69 mV and 11.58 mV, Control vs. aS200pM respectively, no significance. (E) Example current traces for a-SYN aggregates effective concentration treatment: (Ea) Control, (Eb) a-SYN 500 nM (aS500 nM; red), (Ec) washing out after the a-SYN treatment. (F) The current-voltage curve of sodium channel inactivation in the effect concentration treatment, Control (circle, n = 20, from six independent subjects), aS500 nM (square, n = 29, from six independent subjects), Wash out (diamond, n = 19, from six independent subjects). (G) The half-maximal voltage (V) in F, Control, 34.28 mV; aS500 nM, 43.20 mV; Wash out, 39.65 mV, ANOVA (*F*_2, 67_ = 6.32, *p* = 0.003), **p = 0.002 Control vs. aS500 nM. (H) The Boltzmann slope factor in in F, Control, 10.09 mV; aS500 nM, 12.32 mV; Wash out 12.13 mV, no significance. RMP: resting membrane potential.

We next explored the effect of a-SYN aggregates on the ventricular sodium channel inactivation properties (Figure 2). Circulatory level a-SYN aggregates were first examined (Figure 2A-D). The pulse protocol is illustrated in Figure 2Aa. The prepulse voltage at the RMP was first stepped from the RMP to RMP-40 mV. Then depolarizing steps were applied to activate voltage-gated sodium currents (V1 in Figure 2Ab). These depolarizing steps caused first activation then inactivation of sodium channels. Then the voltage step, elicited by the pulse at RMP + 100 mV, was subsequently applied to re-activate the sodium channels (V2 in Figure 2Ab). The amplitude of the sodium current elicited by the second voltage step to RMP + 100 mV was considered to reflect the degree of sodium channel inactivation (Figure 2Ab, arrow mark). The circulatory level a-SYN aggregates did not affect the voltage-gated sodium channel inactivation in the ventricle (Figure 2B-D). Then, the effect of potent concentration a-SYN (500 nM) on ventricular sodium channel inactivation was investigated (Figure 2F-J). The inactivation was described as the value of sodium current normalized to that at the voltage RMP + 0 mV to RMP + 100 mV (Figure 2F), thereby the curves were constructed by plotting from such maximum currents to the initial current (at RMP + 0 mV) where the sodium channel was 100% activated (as of 1.0). The effective concentration a-SYN altered the half-maximum voltage compared to that in control (Figure 2G, *p* = 0.003 Control vs. a-SYN 500 nM (aS500 nM)). However, the slope factor (*k*) was below the significance level. The result suggests that the effective concentration a-SYN aggregates can impact the gating properties of sodium channel inactivation in the ventricle.

### No effect of alpha-synuclein aggregate on the time-recovery from sodium channel inactivation in the ventricle

**Figure 3.**
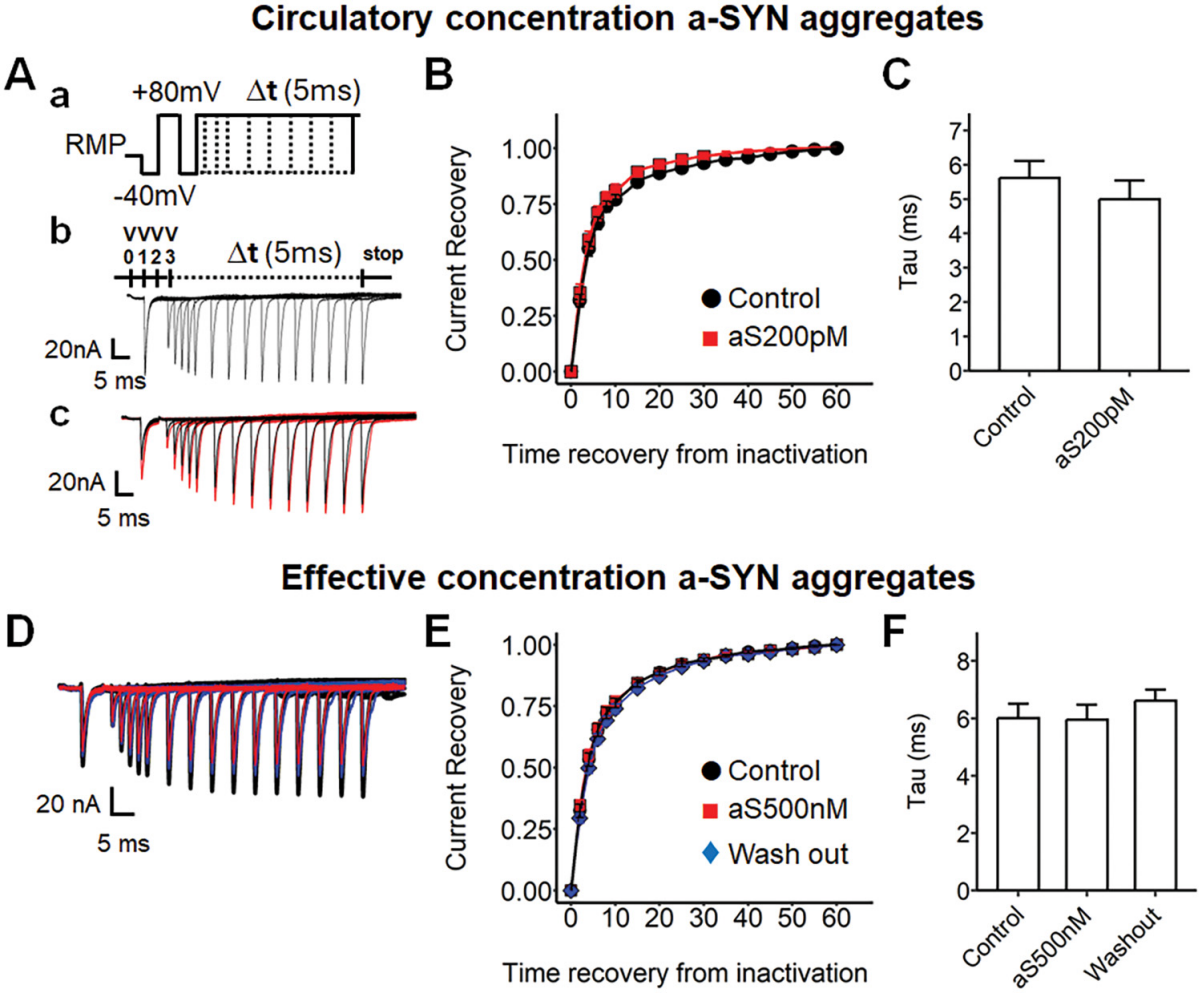
Alpha-synuclein aggregate treatment to the ventricular preparation under the loose patch and the measurement of sodium channel recovery from inactivation following restoration of the membrane potential. (Aa) Pulse protocol for time-increment sodium channel recovery from inactivation, RMP + 0 mV, activation pulse protocol began from the RMP, a 4 ms duration prepulse was applied to the patched area to remove any residual current over the area of the pipette, then 5 ms duration pulses were applied (RMP + 80 mV) to activate ion channels and elicit the voltage-gated currents, then RMP-40 mV was applied to remove the residual voltage, the channels were predicted to inactivate during the phase, then RMP + 80 mV pulse was applied to reactivate the current, the voltage sweeps were imposed with a 1 ms time-increment (Δt*,* 5 ms). (Ab) Example current trace from the pulse protocol, V0, RMP-40 mV prepulse at a 1 ms duration; V1, RMP + 80 mV pulse at a 5 ms duration, V2, RMP-40 mV pulse at a 10 ms duration, V3, RMP + 80 mV pulse at different time intervals; Δt, 5 ms increment via the successive sweeps. (Ac) Example current traces, Control (black), alpha-synuclein (a-SYN) 200 pM treatment (aS200pM, red). (B) The time-voltage curve of sodium channel recovery from inactivation, the maximum *I*_Na_ was plotted against time intervening between the termination of the conditioning and imposition of the test pulse; Control (circle, n = 9, from three independent subjects); aS200pM (square, n = 7, from three independent subjects); each max *I*_Na_ was normalized by the maximum currents (*I*_Na(Max)_) at the termination time-point (65 ms). (C) The time-constant in B, Control, 5.62 mV; aS200pM, 4.99 mV, no significance. (D) Example traces for the effective concentration treatment, Control (black), a-SYN 500 nM treatment (aS500 nM; red), washing out after treatment (blue). (E) The time-voltage curve for effective concentration treatment, Control (circle, n = 14, from six independent subjects), aS500 nM (square, n = 15, from six independent subjects), Wash out (diamond, n = 11, from six independent subjects). (F) Time constant in E, Control, 6.00 mV; aS500 nM, 5.95 mV; Wash out, 6.60 mV, no significance. RMP: resting membrane potential.

The effect of a-SYN on the time-dependence of sodium channel recovery from inactivation was also investigated using the loose patch clamp technique (Figure 3). The pulse protocol was designed to scrutinize the time course of recovery from inactivation in ventricles. The amplitude of each inward sodium current was normalized to the maximum amplitude observed at 100% recovery (recorded at the 65 ms). Circulatory-level concentration of a-SYN did not affect the time-recovery properties from inactivation in the ventricle (Figure 3A-C). Additionally, while effective concentrations of a-SYN aggregates reduced the amplitude of inward sodium current in the ventricle (Figure 3D, red), the normalized current recovery value (relative to 1.0) did not show any significant difference between control and the a-SYN 500 nM (Figure 3E and F).

### Alpha-synuclein aggregates reduced the ventricular potassium currents in the potassium channel activation

**Figure 4.**
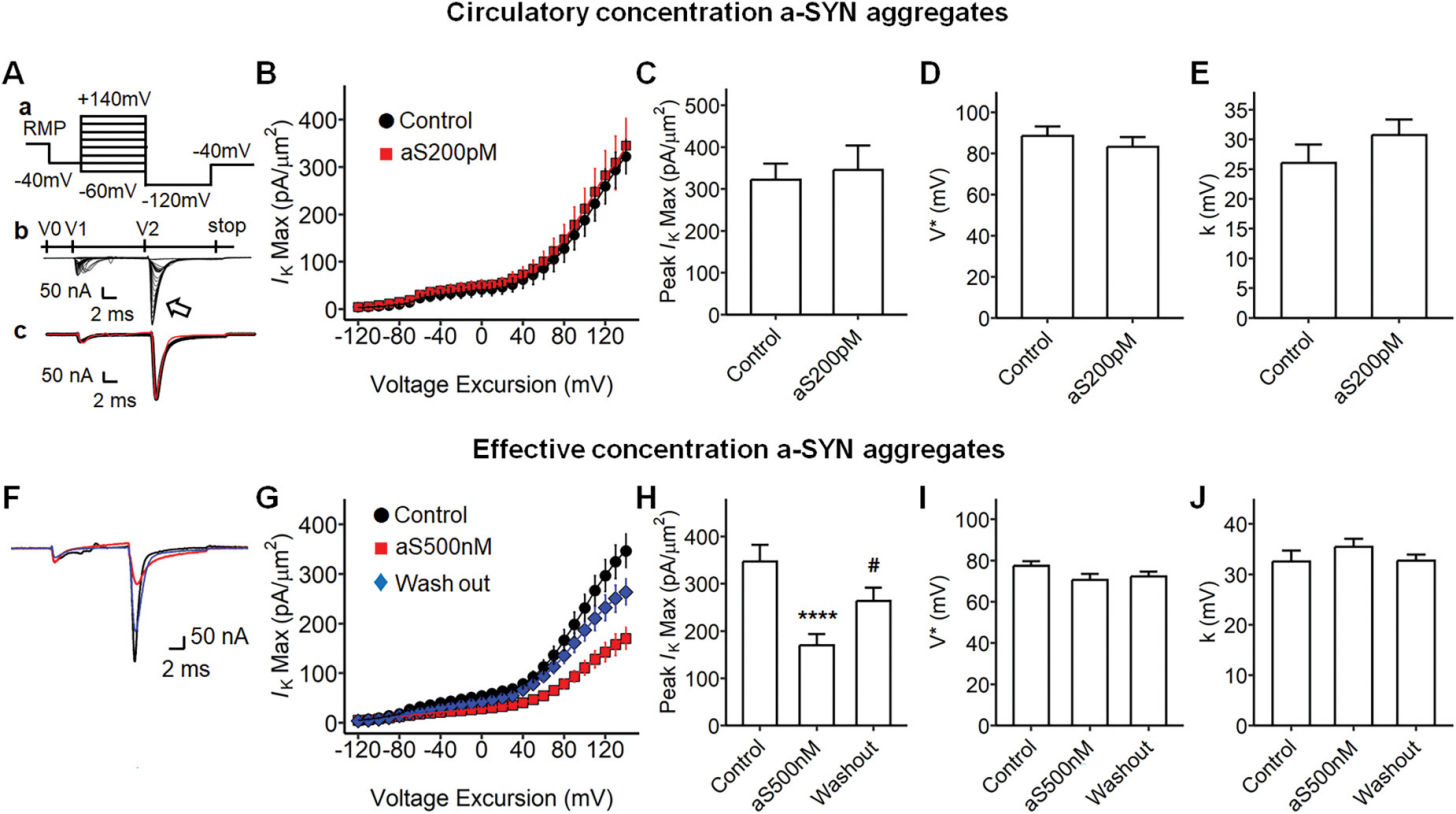
Potassium channel activation properties of alpha-synuclein aggregates-treated ventricular preparations under loose patch. (Aa) Pulse protocol for activating potassium currents for the ventricular preparations, the protocol began at the RMP (resting membrane potential), a 4 ms duration prepulse was applied to the patched area to remove any residual current, then a 10 ms duration step pulses were applied from RMP +140 mV to RMP −60 mV through the 21 successive sweeps, then a 10 ms duration hyperpolarizing step to RMP −120 mV was imposed, then the voltage was stopped at RMP −40 mV. (Ab) A family of current traces from the pulse protocol in Aa, V0, −40 mV prepulse at a 1 ms duration; V1, −10 mV step pulses at a 10 ms; V2, hyperpolarizing pulse at a 20 ms; V3, end pulse at a 30 ms, arrow mark, tail current reflecting potassium current. (Ac) Example current traces, Control (black), alpha-synuclein (a-SYN) aggregates 200 pM in Krebs-Henseleit (KH) solution (aS200pM; red). (B) The current-voltage curve of potassium channel activation, the maximum current (*I*_k(Max)_) was plotted against the voltage excursion; Control (black, n = 9, from three independent subjects), aS200pM (red, n = 8, from three independent subjects). (C) The maximum *I*_k(Max)_ in B, −322.20 mV for Control, −345.60 mV for aS200pM, no statistical significance. (D) The half-maximal voltage (V) from a Boltzmann equation described with B, 88.53 mV for Control, 83.17 mV for aS200pM, no significance. (E) The Boltzmann slope factor (*k*) in B, 26.03 mV for Control, 30.71 mV for aS200pM, no significance. (F) Example current traces for the a-SYN aggregate effect concentration treatment, Control (black), a-SYN 500 nM effective concentration treatment (aS500 nM; red), washing out after treatments (blue). (G) The current-voltage curve of potassium activation with the effective concentration a-SYN, Control (circle, n = 19, from three independent subjects), aS500 nM (square, n = 40, from three independent subjects), Wash out (diamond, n = 31, from three independent subjects). (H) The maximum current (*I*_k(Max)_) in G, −346.88 mV for Control, −170.22 mV for aS500 nM, −263.97 mV for Wash out, ANOVA (F_2, 87_= 9.29, *p* = 0.0002), ****p = 0.0002 Control vs. aS500 nM, #*p* = 0.03 aS500 nM vs. Wash out. (I) The half-maximal voltage (V) in G, 77.51 mV for Control, 70.58 mV for aS500 nM, 72.27 mV for Wash out, no significance. (J) The Boltzmann slope factor (*k*) in G, 32.56 mV for Control, 35.46 mV for aS500 nM, 32.69 mV for Wash out, no significance.

We then determined the effect of extracellular a-SYN on voltage-dependent potassium current in the ventricle (Figure 4). Figure 4Aa illustrates the pulse protocol to activate voltage-gated potassium current in the ventricle. After 10 ms duration of pre-pulse, the procedure evokes a depolarization step to voltage between RMP-60 mV to RMP +140 mV which elicited sodium channel activation, followed by its inactivation (V1 in Figure 4Ab). This was then followed by a subsequent hyperpolarizing step (RMP-120 mV). The hyperpolarizing step resulted in tail currents, reflecting the preceding voltage-gated potassium current activation in the ventricle (V2 in Figure 4Ab, arrow mark). We investigated circulatory-level of a-SYN in the ventricle; however, there was no significant difference between the control and the treatment group (Figure 4B-E). We then examined the effect of potent concentration a-SYN in potassium channel inactivation (Figure 4F-J). Interestingly, we observed that the potassium current decreased in a-SYN 500 nM treatment (Figure 4F and G, *p* = 0.0002 Control vs. aS500 nM; *p* = 0.03 aS500 nM vs. Wash out) which recovered on wash out. The maximum *I*_K(Max)_ in a-SYN 500 nM was reduced compared to that in control (Figure 4H). We analyzed the kinetics of the potassium channel activation but no significant alterations in half-maximal voltage or in slope factor (Figure 4I and J) were observed. The potent concentration of a-SYN altered the maximal potassium current; however, it did not affect the kinetics of the channel activation.

### Potassium rectification current was decreased by alpha-synuclein treatment

**Figure 5.**
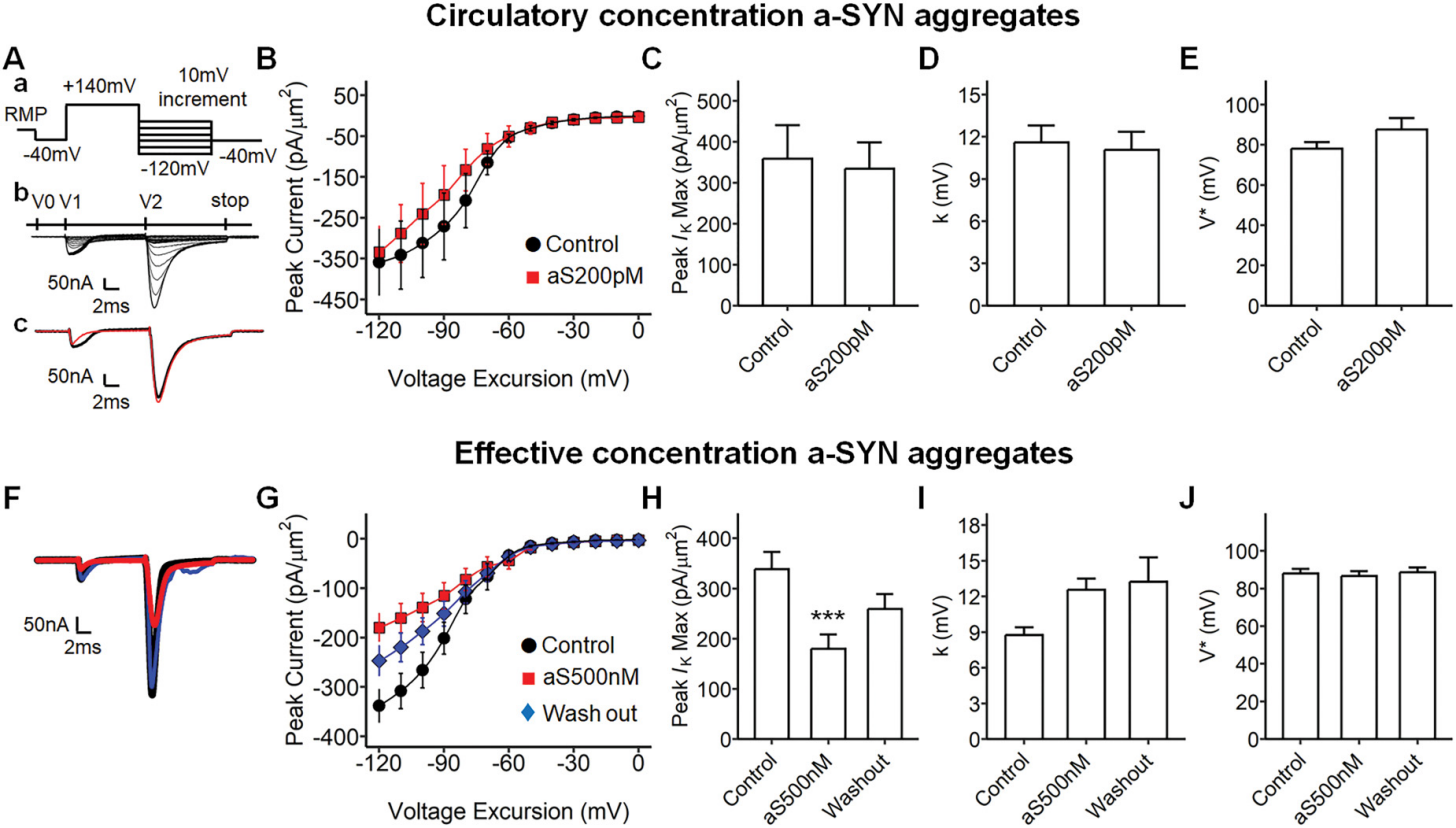
Potassium channel reversal properties in alpha-synuclein aggregate treated ventricular preparations. (Aa) Step pulse protocol to rectify potassium current, pulse protocol began at RMP (resting membrane potential), a 4 ms duration prepulse was applied to remove residual current, then a 10 ms duration voltage was applied at RMP +140 mV, then a 10 ms duration voltage sweep from RMP −120 mV to RMP +50 mV, then the stop voltage sweep at RMP −40 mV was applied. (Ab) A family of typical current traces from the pulse protocol in Aa. V0, −40 mV prepulse at a 1 ms duration; V1, 10 mV step pulses at a 10 ms; V2, hyperpolarizing pulse at a 20 ms; V3, stop pulse at a 30 ms. Ac, the example current traces, Control (black), alpha-synuclein (a-SYN) aggregates 200 pM treatment (aS200 pM, red). (B) The current-voltage curve of potassium channel reversal, the maximum current (*I*_k(Max))_ was plotted against the voltage excursion; Control, baseline recording with Krebs-Henseleit solution (KH, circle black, n = 9, from three independent subjects); aS200pM (square red, n = 8, from three independent subjects). (C) The maximum *I*_K(Max)_ in B, −345.40 mV for Control, −322.20 mV for aS200pM, no significance. (D) The Boltzmann slope factor (*k*) in B, 11.95 mV for *I*_K_ in Control, 11.07 mV for *I*_K_ in aS200pM, no significance. (E) The half-maximal voltage (V) from a Boltzmann equation described with B, 78.07 mV for *I*_K_ in Control, 87.56 mV for *I*_K_ in aS200pM, no significance. (F) Example current traces in the a-SYN 500 nM effective concentration treatment, Control (black), a-SYN 500 nM treatment (aS500 nM, red), washing out with Krebs-Henseleit after treatments (blue). (G) The current-voltage curve of potassium channel reversal in the effect concentration alpha-synuclein treatment, Control (circle, n = 19, from six independent subjects), aS500 nM (square, n = 33, from six independent subjects), Wash out (diamond, n = 29, from six independent subjects), no significance. (H) The maximum *I*_K_ in G, −376.35 mV for Control, −179.70 mV for aS500 nM, −259.33 mV for Wash out, ANOVA (F_2, 77_ = 6.02, ***p = 0.002), ***p = 0.002 Control vs. aS500 nM. (I) The Boltzmann slope factor (*k*) in G, 8.19 mV in *I*_K_ for Control, 12.56 mV in *I*_K_ for aS500 nM, 13.24 mV in *I*_K_ for Wash out, no significance. (J) The half-maximal voltage (V) in G, 85.87 mV for Control, 86.67 mV for aS500 nM, 88.66 mV for Wash out, no significance. RMP: resting membrane potential.

We next explored the effect of a-SYN aggregates on the ventricular potassium rectifying current (Figure 5). Figure 5A illustrates the pulse protocol and example current traces from investigations of potassium current rectification. The first voltage step (V1 in Figure 5Ab) initially elicited an activation of inward sodium current, followed by its inactivation, and then a rectifying potassium current activation (V2 in Figure 5Ab). The circulatory level a-SYN aggregates did not affect the potassium rectifying current activation properties in the ventricle (Figure 5B-E), but the effective concentration significantly decreased the amplitude of peak rectifying current (Figure 5G and H, *p* = 0.002 Control vs. aS500 nM). The a-SYN showed consistently smaller potassium currents than did the control preparations. However, the channel gating properties were not affected by the a-SYN treatment (Figure 5I and J).

### Exogenous alpha-synuclein aggregate did not affect sodium and potassium currents in stellate ganglia under the loose patch clamp

**Figure 6.**
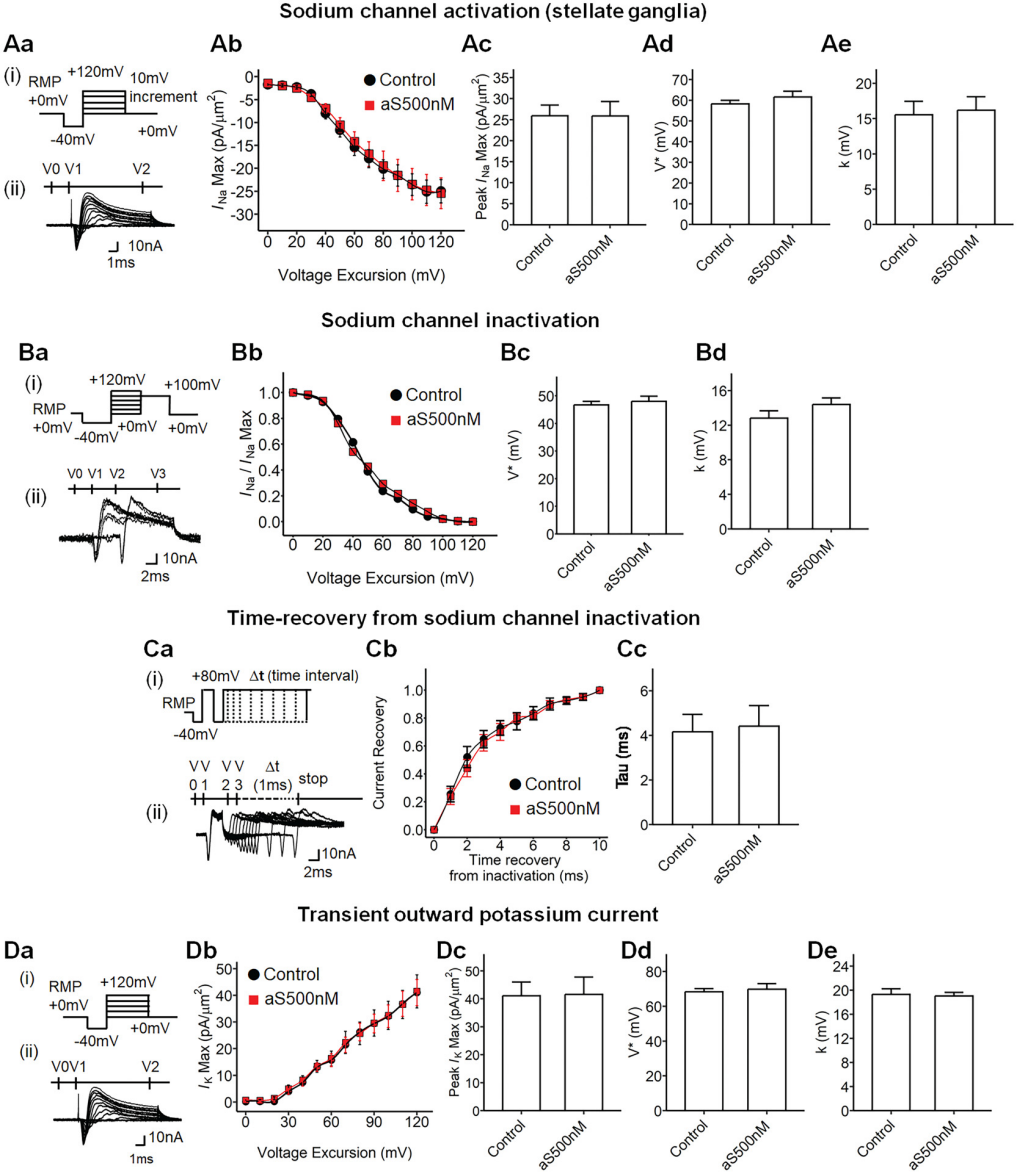
Alpha-synuclein aggregate effective concentration treatment to the stellate ganglia. (A) Sodium current activation properties in stellate ganglia with alpha-synuclein (a-SYN) treatment. (Aa) Step pulse protocol to activate sodium currents for stellate ganglia (i), a family of current traces (ii); (Ab) Current-voltage curve for sodium current activation in stellate ganglia, Control (black, n = 10), a-SYN 500 nM treatment (aS500 nM; red, n = 10); (Ac) Maximum *I*_Na(Max)_ current in Ab; (Ad) Half-maximal voltage in Ab; (Ae) The Boltzmann slope factor in Ab, no significance. (B) Sodium current inactivation properties in stellate ganglia with a-SYN treatment; (Ba) Pulse-protocol for sodium current inactivation (i), example current trace (ii); (Bb) The current-voltage curve for sodium channel inactivation, each current was normalized to its greatest value at RMP +0 mV; Control (black, n = 12), aS500 nM (red, n = 10); (Bc) Half-maximal voltage in Bb; (Bd) The slope factor in Bb, no significance. (C) Time-recovery from inactivation in stellate ganglia with a-SYN treatment; (Ca) Pulse protocol (i), example current traces (ii); (Cb) The time-voltage curve of sodium channel recovery from inactivation, Control (black, n = 10), aS500 nM (red, n = 10), each inward sodium current was normalized by the maximum value at 10 ms; (Cc) The time-constant tau (τ) in Cb, no significance. (D) Transient outward potassium current properties in stellate ganglia with a-SYN treatment; (Da) Pulse protocol for activating transient outward potassium current (i), example current trace (ii); (Db) Current-voltage curve, Control (black, n = 12), aS500 nM (red, n = 10); (Dc) Maximum current (*I*_K(Max)_) in Db; (Dd) The half-maximal voltage (V*) in Db; (De) The slope factor in Db, no significance. The patch clamp data for the stellate ganglia tissue was recorded from three independent subjects. RMP: resting membrane potential.

Lastly, a-SYN aggregates were superfused in the stellate gangla and the effect on sodium and potassium currents were again investigated using the loose patch clamp as previusly described.^36^ We here examined both circulatory-level (200 pM; Supplemental Figures 7 and 8) and potent concentrations effect on the stellate ganglia tissue (500 nM; Figure 6); inward sodium currents (Figure 6A-C), and transient outward potassium currents (Figure 6D) were recorded. At both concentrations, the a-SYN aggregates did not alter the sodium and potassium currents in the stellate ganglia. The incubation time for a-SYN was also extended to 45 min to observe long-term exposure effect. However, there were no significant differences observed between the control and a-SYN treatments.

## Discussion

The present study explored a-SYN aggregate-mediated electrophysiological alterations in ventricular myocardium. Recent research has indicated that the heart can develop alpha-synucleinopathy.^29–31^ In addition, PD has also been associated with cardiac electrophysiological alterations, as reflected by electrograms in PD patients.^26^ However, there is a lack of studies that explore the link between alpha-synucleinopathy and changes in cardiac electrophysiology. The present study demonstrated for the first time that extracellular a-SYN aggregates can have an inhibitory effect on the modulation of ventricular sodium and potassium currents in the myocardium. Additionally, we observed that the a-SYN aggregates only affected the ventricular preparation and did not affect the SG, implying a tissue-specific sensitivity to a-SYN between the heart and SG.

Foremost, high-dose a-SYN aggregates reduced sodium and potassium currents in the ventricular myocardium. The a-SYN aggregates altered the maximum inward sodium currents in sodium activation, the gating kinetics of sodium channel inactivation, and reduced the maximum outward activation and rectifying currents. We propose that a-SYN may affect cardiac tissue either via membrane interaction or by modulating cardiac ion channels. The reversibility of the inhibition induced by a-SYN during wash out supports both of these hypotheses. Since a-SYN aggregates non-specifically affect both sodium and potassium currents, the inhibitory effect may be elicited by alterations in the broad cellular status, such as cell stress or membrane interactions, rather than by regulating ionic channels. In this case, membrane-bound a-SYN might form a pore, which acted as an ionic gate,^25^ resulting in alteration of transmembrane potential in the cardiac membranes.^24^ The loose patch clamp technique measures the transmembrane current flow into an extracellular electrode opposed to the myocardial tissue surface. Thus, if the a-SYN aggregates altered the membrane on the ventricular myocardium, the currents through ion channels might be affected accordingly. Nevertheless, given that the same concentration of a-SYN did not affect the SG tissue, it cannot be concluded that this effect is broadly applicable across cell types. Instead, it appears that cardiac tissue exhibits greater sensitivity to a-SYN compared to SG. Furthermore, there is a possibility that a-SYN may directly interact with cardiac ion channels, thereby altering sodium and potassium currents in the myocardium. Alterations in voltage dependence (V*) and slope factor (*k*) in the ventricular myocardium may reflect a modulation of cardiac sodium channel gating properties in response to a-SYN. Although, no direct interaction between a-SYN and cardiac ion channels has been reported to date, a-SYN is not devoid of channel-binding capacity; it has been shown to interact with ATP-sensitive potassium channel (K_ATP_) in pancreatic beta cells and with sarcoendoplasmic reticulum calcium-ATPase in neuronal cells.^42,43^ In addition, the current observed may have been affected by other ion channels, such as sodium-calcium-exchanger, or by intracellular ionic dynamics.^20,42^ In neuronal cells, a-SYN has been shown to disrupt cytoplasmic calcium homeostasis; however, the mechanism underlying its cytoplasmic propagation remains unclear.^20,42,44^

Secondly, only the higher concentration of a-SYN aggregates altered the cardiac ionic currents, whereas the lower concentration had no significant effect, suggesting a-SYN appears to exert differential effects depending on its concentration. The plasma circulating-level concentration in the present study was adapted from Kim et al. (2022), reflecting a predicted pathological plasma concentration of a-SYN as in *in vivo* PD model.^40^ However, the circulating-level concentration did not significantly affect the ionic currents in the ventricle and SG *ex vivo*. On the contrary, the higher concentration of a-SYN significantly altered cardiac ionic currents. An earlier study reported that the membrane binding affinity of a-SYN was dose-dependent.^45^ Therefore, the higher concentration of a-SYN might elicit the effect due to its stronger interaction with a-SYN aggregates on the cardiac tissue.^30,31^ Studies on cardiac a-SYN have reported the deposition of a-SYN aggregates within epicardial neurons and adipose tissues;^30,31^ however, the local circulating concentration of a-SYN within the heart remains undetermined and may differ from its levels in systemic circulation. Nonetheless, given that cytosolic a-SYN aggregates can be released into the extracellular space via non-classical exocytosis or exosome,^46–49^ it is possible that cardiac tissue may be exposed to locally elevated concentrations of a-SYN aggregates in individuals with cardiac alpha-synucleinopathy. The present study presumed that the concentration of a-SYN remained constant at both low and effective concentrations. However, in a physiological biosystem, more complex mechanisms of concentration regulation are likely to occur. Therefore, even if a-SYN impacts the heart, it remains uncertain whether this effect is sustained chronically. If the elevated concentration is transient and followed by a wash out, the effect may be reversible, as observed in our experimental findings.

Finally, the a-SYN did not affect the currents in SG, raising the possibility that ganglionic cells may respond differently to a-SYN aggregates compared to cardiomyocytes. We technically verified that the perineural barrier that insulates the ganglionic cells was removed prior to patch clamping the tissues (Supplemental Figure 1). Therefore, the a-SYN aggregates reached the ganglionic cells, but still SG did not respond to a-SYN aggregates. This finding contrasts with studies by Ferreira et al. (2017), which suggested that 500 nM a-SYN aggregates affected postsynaptic activity via interaction with NMDAR2B receptors in hippocampal neurons.^21,50^ This discrepancy is likely attributable to differences in NMDA receptors and their associated downregulating pathways between the CNS and the stellate ganglia. Furthermore, given that the a-SYN aggregates we tested showed no effect at both lower and higher concentrations, it is unlikely that extracellular circulating a-SYN would exert significant effects under physiological conditions. However, experimental evidence suggests that exposure to extreme concentrations of a-SYN (2 μM) *in vitro* can impact neuronal excitation via membrane perturbation.^20^ Moreover, this experiment assessed the toxicity of a-SYN based solely on the assumption that plasma-circulating a-SYN is present in the heart and SG, and the potential risk associated with cytoplasmic a-SYN aggregates cannot be overlooked.

In summary, we found that a-SYN aggregates can alter electrophysiology in the ventricular myocardium. This finding may explain the cardiac electrophysiological abnormalities observed in the PD patients with cardiac alpha-synucleinopathy, potentially leading to sudden arrhythmic death. Cardiac alpha-synucleinopathy is an emerging area of research, and although its prevalence remains unknown, its potential association with SCD is of critical importance and warrants attention.^29^ Our study highlights a direct association between a-SYN and cardiac electrophysiology. Further research aimed at elucidating the mechanism by which a-SYN aggregates affect the ventricles will be essential for developing future treatments targeting the cardiac manifestations of PD.

## Supplemental Material

sj-docx-1-pkn-10.1177_1877718X251365239 - Supplemental material for In situ exogenous alpha-synuclein aggregates inhibit murine ventricular voltage-gated inward sodium and outward potassium currents Supplemental material, sj-docx-1-pkn-10.1177_1877718X251365239 for In situ exogenous alpha-synuclein aggregates inhibit murine ventricular voltage-gated inward sodium and outward potassium currents by Bonn Lee, Shiraz Ahmad, Charlotte E Edling, Hugh R Matthews, Christopher L-H Huang, Fiona EN LeBeau and Kamalan Jeevaratnam in Journal of Parkinson's Disease
