# Supplementary material for: In situ exogenous alpha-synuclein aggregates inhibit murine ventricular voltage-gated inward sodium and outward potassium currents
Source: J Parkinsons Dis. 2025 Aug 28:1877718X251365239. Online ahead of print. doi: 10.1177/1877718X251365239 (PMC13347525; doi:10.1177/1877718X251365239)
Supplement: Supplementary file 1 [file sj-docx-1-pkn-10.1177_1877718X251365239.docx]

**Supplemental Material**

**In situ exogenous alpha-synuclein aggregates inhibit murine ventricular voltage-gated inward sodium and outward potassium currents**

**Supplemental Figure 1. Enzymatic removal of the neural barriers of the stellate ganglia.** The epineurium and perineural cells were removed by enzymatic digestion using collagenase P. The stellate ganglia were incubated with 2 mg/mL collagenase P in physiological solution at 36°C. (A) Masson’s trichrome staining was performed to locate the epineurium of the stellate ganglion. A collagen-rich area was stained blue (indicated by the red arrow). (B) Hematoxylin and eosin staining (H&E). Perineural fibroblasts were observed in the naïve tissue (left, indicated by the black arrow), while the cell layer was not observed in the tissue after the incubation (right). Naive, stellate ganglia without enzymatic digestion (left); Collagenase, stellate ganglion after collagenase incubation (right). H&E staining revealed the perineural fibroblast layer surrounding the stellate ganglion (black arrow). Scale bar, 10 mm. (C) The current traces from stellate ganglia with and without collagenase incubation. (a) voltage clamp protocols, RMP, resting membrane potential; (b) a current trace recorded with the stellate ganglia without collagenase incubation, no current was observed in the naïve tissue; (c) a current trace from the stellate ganglion with collagenase incubation. An inward sodium current was observed at 50 ms, followed by an outward potassium current.

**Supplemental Figure 2. Random peptide treatment to the ventricular preparation and the measurement of its sodium channel activation properties.** (A) Step pulse protocol to activate sodium currents for the ventricular preparations (a), RMP resting membrane potential, activation pulse protocol began from the RMP, a 4 ms duration prepulse was applied to the patched area to remove any residual current over the area of the pipette, then 10 ms step pulses were applied to elicit the voltage-gated currents negative 40 mV from the RMP (RMP -40 mV) and through to positive 120 mV from the RMP (RMP +120 mV); a family of current traces from the pulse protocol in (b). V0, -40 mV prepulse at a 1 ms duration; V1, 10 mV step pulses at a 5 ms duration. (B) The current-voltage curve of sodium activation protocol for ventricle preparation under loose patch, *I*_Na(Max)_ was plotted against the voltage excursion; Control, baseline recording with Krebs-Henseleit solution (KH, circle, n = 6, from three independent subjects); PEP, recording with 5 mM random peptide treatment in KH (triangle, n = 7); The maximum current (*I*_Na(Max)_) increased along the voltage excursion up to the *I*_Na(Max)_ and the activation decayed when it gets its greatest value. (C) The maximum current (*I*_Na(Max)_) of ventricle preparations for control and random peptide, -68.370 mV for KH control, -71.442 mV for the KH with random peptide, no statistical significance. (D) The Boltzmann slope factor (*k*) for ventricle preparation in KH control and KH with random peptide. The sodium activation current was fitted with a Boltzmann function, *k* of 5.381mV for *I*_Na_ in control, 6.246 mV for *I*_Na_ in KH with random peptide, no significance. (E) The half-maximal voltage (*V**) from a Boltzmann equation described with B, 29.10 mV for *I*_Na_ in KH control, 30.767 mV for *I*_Na_ in KH with random peptide, no significance.

**Supplemental Figure 3. Random peptide treatment to the ventricular preparation and the measurement of its sodium channel inactivation properties.** (A) Step pulse protocol (a), RMP resting membrane potential, activation pulse protocol began from the RMP, a 4 ms duration prepulse was applied to the patched area to remove any residual current over the area of the pipette, then 10 ms step pulses were applied to elicit the voltage-gated currents negative 40 mV from the RMP (RMP -40 mV) and through to positive 120 mV from the RMP (RMP +120 mV), finally all sweeps was stopped to a RMP +100 mV; a family of current traces from the sodium channel inactivation protocol in a (b), arrow mark, the maximum inward sodium current (*I*_Na(Max)_) after the activating pulse, the current get smaller along the step as the sodium channels became refractory to the pulse (RMP +100 mV) after the activating pulse; V0, -40 mV prepulse at a 1 ms duration; V1, 10 mV step pulses at a 5 ms duration, V2, 100 mV depolarizing pulse at a 10 ms duration. (B) The current-voltage curve of sodium channel inactivation under loose patch, each value of sodium current (A-b, arrow marked) was plotted against the voltage excursion (*I*_Na(Max)_), each value was normalized to its greatest value at RMP + 0 mV; Control, baseline recording with Krebs-Henseleit solution (KH, circle, n = 6, from three independent subjects); PEP, recording with 5 mM random peptide treatment in KH (triangle, n = 7); The *I*_Na(Max)_ increased along the voltage excursion up to the *I*_Na(Max)_ and the activation decayed when it gets its greatest value. (C) The Boltzmann slope factor (*k*) for ventricle preparation in KH control and KH with random peptide. The sodium activation current was fitted with a Boltzmann function, *k* of 15.973 mV for *I*_Na_ in KH control, 9.925 mV for *I*_Na_ in KH with random peptide, no significance. (C) The half-maximal voltage (V) from a Boltzmann equation described with B, -42.307 mV for *I*_Na_ in KH control, -35.740 mV for *I*_Na_ in KH with random peptide, no significance.

**Supplemental Figure 4. Random peptide treatment to the ventricular preparation and the measurement of its sodium channel recovery from inactivation following restoration of the membrane potential.** (A) The pulse protocol (a), RMP resting membrane potential, activation pulse protocol began from the RMP, a 4 ms duration prepulse was applied to the patched area to remove any residual current over the area of the pipette, then 10 ms step pulses were applied to elicit the voltage-gated currents negative 40 mV from the RMP (RMP -40 mV) and to positive 120 mV from the RMP (RMP +120 mV), then RMP +100 mV was applied; a family of current traces from the sodium channel inactivation protocol in a (b). V0, -40 mV prepulse at a 1 ms duration; V1, RMP +80 mV pulse at a 5 ms duration, V2, RMP-40 mV pulse at a 10 ms duration, V3, RMP +80 mV pulse at a different time intervals, Δ*t*, between 5 ms (V3) and 65 ms (V3’) increment via the 12 successive sweeps making up the protocol. (B) The time-voltage curve of sodium channel recovery from inactivation, the maximum current (*I*_Na(Max)_) was plotted against time intervening between the termination of the conditioning and imposition of the test pulse; Control, baseline recording in Krebs-Henseleit solution (KH, circle mark, n = 6, from three independent subjects); PEP, recording in KH with 5 mM random peptide (triangle, n = 6); each maximum current was normalized to its greatest value at the final step (65 ms). (C) The time-constant for KH control and KH with random peptide under the loose patch, 14.814 ms for *I*_Na_ in KH control, 15.905 ms for *I*_Na_ in KH with random peptide, no significance.

**Supplemental Figure 5. Random peptide treatment to the ventricular preparation and the measurement of its potassium channel activation properties.** (A) Step pulse protocol to activate potassium channel for the ventricular preparations (a), RMP, resting membrane potential; activation pulse protocol began from the RMP, a 4 ms duration prepulse was applied to the patched area to remove any residual current, then a 10 ms duration step pulses were applied from RMP +140 mV to RMP -60 mV through the 21 sweeps, then a 10 ms duration hyperpolarizing step to RMP -120 mV was imposed, finally the membrane potential was turn back to RMP -40 mV; a family of current traces from the pulse protocol in a (b). V0, -40 mV prepulse at a 1 ms duration; V1, -10 mV step pulses at a 10 ms; V2, hyperpolarizing pulse at a 20 ms; V3, end pulse at a 30 ms. (B) The current-voltage curve of potassium channel activation protocol for ventricle preparation under the loose patch, the maximum current (*I*_K(Max)_) was plotted against the voltage excursion; Control, baseline recording with Krebs-Henseleit solution (KH, circle mark, n = 8, from three independent subjects); PEP, recording with random peptide treatment in KH (triangle, n = 10). (C) The maximum current (*I*_K(Max)_) of ventricle preparations for control and random peptide, 258.63 mV for KH control, 254.52 mV for the KH with random peptide, no statistical significance (*t*_15.283_ = -0.055, *p* = 0.956). (D) The Boltzmann slope factor (*k*) for ventricle preparation in KH control and KH with 5 mM random peptide. The sodium activation current was fitted with a Boltzmann function, *k* of 26.32 mV for *I*_K_ in control, 25.45 mV for *I*_K_ in KH with random peptide, no significance (*t*_13.447_ = -0.031, *p* = 0.76). (E) The half-maximal voltage (*V**) from a Boltzmann equation described with B, 90.94 mV for *I*_K_ in KH control, 88.52 mV for *I*_K_ in KH with random peptide, no significance (*t*_15.897_ = 0.391, *p* = 0.701).

**Supplemental Figure 6. Random peptide treatment to the ventricular preparation and the measurement of its potassium current rectification properties.** (A) Step pulse protocol to rectify potassium current (a), RMP, resting membrane potential; activation pulse protocol began from the RMP, a 4 ms duration prepulse was applied to the patched area to remove any residual current, then a 10 ms duration fixed voltage at RMP +140 mV were applied, then a 10 ms duration voltage sweep from RMP -120 mV to RMP +50 mV followed, finally the voltage sweep of RMP -40 mV was applied; a family of typical current traces from the pulse protocol in a (b). V0, -40 mV prepulse at a 1 ms duration; V1, 10 mV step pulses at a 10 ms; V2, hyperpolarising pulse at a 20 ms; V3, termination pulse at a 30 ms. (B) The current-voltage curve of potassium channel activation protocol for ventricle preparation under the loose patch, *I*_K(Max)_ of each value of potassium current was plotted against the voltage excursion; Control, baseline recording with Krebs-Henseleit solution (KH, circle, n = 7, from three independent subjects); PEP, recording with random peptide treatment in KH (triangle, n = 6, from three independent subjects). (C) The greatest value of *I*_K(Max)_ in B, -200.39 mV for KH control, -248.35 mV for the KH with random peptide, no statistical significance (*t*_7.65_ = 0.72, *p* = 0.49). (D) The Boltzmann slope factor (*k*) for ventricle preparation in KH control and KH with 5 mM random peptide. The potassium activation current was fitted with a Boltzmann function, *k* of 11.65 mV for *I*_K_ in KH control, 12.73 mV for *I*_K_ in KH with random peptide, no significance (*t*_9.41_ = -0.56, *p* = 0.58). (E) The half-maximal voltage (*V**) from a Boltzmann equation described with B, -85.31 mV for *I*_K_ in KH control, -84.82 mV for *I*_K_ in KH with random peptide, no significance (*t*_9.05_ = -0.06, *p* = 0.94).

**Supplemental Figure 7. Low concentration alpha-synuclein aggregate treatment to the stellate ganglia preparation under loose patch and the measurement of its sodium channel activation properties.** (A) Step pulse protocol to activate sodium currents for the ventricular preparations (a), a family of current traces from the pulse protocol in a (b), example traces (c), black trace, control, red trace, alpha-synuclein 200 pM. (B) Current-voltage curve, baseline recording in physiological solution (Control; n = 10, from three different mice), alpha-synuclein 200 pM (aS200pM, n=11, from three different mice). (C) The peak *I*_Na(Max)_ in B. (D) The Boltzmann slope factor in B. (E) The half-maximal voltage in B, no significant Control vs. aS200pM.

**Supplemental Figure 8. Circulatory-level concentration alpha-synuclein aggregates treatment to the stellate ganglia and its sodium channel inactivation properties.** (A) Step pulse protocol (a), RMP resting membrane potential, activation pulse protocol began from the RMP, a 4 ms duration prepulse was applied to the patched area to remove any residual current over the area of the pipette, then 10 ms step pulses were applied to elicit the voltage-gated currents negative 40 mV from the RMP (RMP -40 mV) and to positive 120 mV from the RMP (RMP +120 mV), finally all sweeps was stopped to a RMP +100 mV; a family of current traces from the sodium channel inactivation protocol in a (b), arrow mark, the inward sodium current after the channel inactivation, the size of the currents get smaller along the step voltage increment (from RMP +0 mv to +120 mV) as the sodium channels became refractory after their channel activation; V0, -40 mV prepulse at a 1 ms duration; V1, 10 mV step pulses at a 5 ms duration, V2, 100 mV depolarising pulse at a 10 ms duration. The example current traces for control and alpha-synuclein (a-SYN) 200pM (c), black trace, control with the physiological solution, red trace, a-SYN 200 pM. (B) The current-voltage curve of sodium channel inactivation. The value of sodium current was normalized to its greatest value at RMP +0 mV, baseline recording in KH (Control, circle, n = 10, from three independent subjects) vs. aS200pM (square, n = 11, from three independent subjects). (C) The half-maximal voltage (*V**) from a Boltzmann equation described with B, no significance. (D) The Boltzmann slope factor (*k*) in B, no significance.
